# Investigating the Role of PPARβ/δ in Retinal Vascular Remodeling Using *Pparβ*/*δ*-Deficient Mice

**DOI:** 10.3390/ijms21124403

**Published:** 2020-06-20

**Authors:** Sze Yuan Ho, Yuet Ping Kwan, Beiying Qiu, Alison Tan, Hannah Louise Murray, Veluchamy Amutha Barathi, Nguan Soon Tan, Chui Ming Gemmy Cheung, Tien Yin Wong, Walter Wahli, Xiaomeng Wang

**Affiliations:** 1 Lee Kong Chian School of Medicine, Nanyang Technological University Singapore, 59 Nanyang Drive, Singapore 636921, Singapore; hosz0002@e.ntu.edu.sg (S.Y.H.); kwanyuetping@ntu.edu.sg (Y.P.K.); nstan@ntu.edu.sg (N.S.T.); 2 Institute of Molecular and Cell Biology, Agency for Science, Technology and Research (A*STAR), Proteos, 61 Biopolis Dr., Singapore 138673, Singapore; byqiu@imcb.a-star.edu.sg (B.Q.); cltan@imcb.a-star.edu.sg (A.T.); murrayh@imcb.a-star.edu.sg (H.L.M.); 3 Singapore Eye Research Institute, The Academia, 20 College Road, Discovery Tower Level 6, Singapore 169856, Singapore; amutha.b.veluchamy@seri.com.sg; 4 Academic Clinical Program in Ophthalmology, DUKE-NUS Graduate School of Medicine, Singapore 169857, Singapore; 5 Department of Ophthalmology, Yong Loo Lin School of Medicine, National University of Singapore, 1E Kent Ridge Road, NUHS Tower Block, Level 7, Singapore 119228, Singapore; 6 School of Biological Sciences, Nanyang Technological University Singapore, 60 Nanyang Drive, Singapore 637551, Singapore; 7 Center for Integrative Genomics, Université de Lausanne, Le Génopode, CH-1015 Lausanne, Switzerland; 8 UMR 1331, Institut National de la Recherche Agronomique (INRA), Toxalim (Research Centre in Food Toxicology), 180, chemin de Tournefeuille, 1331 Toulouse, France; 9 Institute of Ophthalmology, University College London, 11-43 Bath Street, London EC1V 9EL, UK

**Keywords:** PPARβ/δ, angiogenesis, blood vessel remodeling, pericytes, vessel caliber, arteriovenous crossover

## Abstract

Peroxisome proliferator-activated receptor (PPAR)β/δ is a member of the nuclear receptor superfamily of transcription factors, which plays fundamental roles in cell proliferation and differentiation, inflammation, adipogenesis, and energy homeostasis. Previous studies demonstrated a reduced choroidal neovascularization (CNV) in *Pparβ*/*δ*-deficient mice. However, PPARβ/δ’s role in physiological blood vessel formation and vessel remodeling in the retina has yet to be established. Our study showed that PPARβ/δ is specifically required for disordered blood vessel formation in the retina. We further demonstrated an increased arteriovenous crossover and wider venous caliber in *Pparβ*/*δ*-haplodeficient mice. In summary, these results indicated a critical role of PPARβ/δ in pathological angiogenesis and blood vessel remodeling in the retina.

## 1. Introduction

Over the last two decades, agents targeting vascular endothelial growth factor (VEGF) have revolutionized the treatment for ocular angiogenic diseases [1,2,3], including proliferative diabetic retinopathy (PDR), diabetic macular edema (DME), neovascular age-related macular degeneration (nAMD), and retinal vein occlusion (RVO). Despite the clear efficacy of intervention, a substantial number of patients do not respond to the treatment or may develop resistance over time [4], which is likely due to the compensatory activation of alternative angiogenic pathways [5,6]. Long-term treatment with anti-VEGF drugs also raises concerns regarding potential adverse side effects [7,8] due to the essential role of VEGF in physiological angiogenesis, such as during wound repair [9], and neuro-protection [10]. Furthermore, anti-VEGF treatment has been reported to cause accelerated fibrosis with up to 50% nAMD patients developing sub-retinal fibrosis after 2 years of anti-VEGF treatment [11]. Hence, effective treatments for ocular angiogenic diseases remain a significant un-met medical need. Great efforts have been made to develop alternative or complementary treatment strategies to current anti-VEGF therapeutics [12]. 

Emerging evidence suggests that targeting cell metabolism may offer an effective strategy to control abnormal blood vessel formation [13]. Peroxisome proliferator-activated receptors (PPARs) are ligand-activated nuclear receptors. Besides their well-established roles in lipid and carbohydrate metabolism [14,15,16], PPARs are found to be expressed in endothelial cells (ECs) and control new blood vessel formation [17]. Among all three PPAR isotypes, namely PPARα, PPARβ/δ, and PPARγ, the role of PPARβ/δ in angiogenesis is less well characterized. PPARβ/δ activation by a selective agonist GW501516 was previously reported to promote EC activation and angiogenesis in Matrigel plug assay in vivo [18]. Manipulating PPARβ/δ’s action using a specific agonist or antagonist has also been shown to affect retinal blood vessel formation in rats [19]. Furthermore, PPARβ/δ antagonist treatment is protective against the VEGF-induced retinal vascular permeability [20]. Consistent with these observations, a reduced choroidal neovascularization (CNV) has been observed in PPARβ/δ-deficient mice subjected to laser-induced CNV [21]. Nevertheless, the specific roles of PPARβ/δ in physiological retinal angiogenesis, as well as in retinal blood vessel remodeling, have yet to be established. In this study, we carried out a detailed characterization of retinal vasculature in *Pparβ*/*δ*-haplodeficient mice. Although reduced PPARβ/δ expression does not affect physiological angiogenesis, our study revealed critical roles of PPARβ/δ in the formation of tortuous, chaotic, and disorganized blood vessel and vascular remodeling in the retina. We focused mainly on the characterization of the retinal phenotype of the *Pparβ*/*δ*-haplodeficient mice because their phenotype best corresponds to human diseases, such as branch RVO.

## 2. Results

### 2.1. PPARβ/δ Is Highly Expressed in the Mouse Retina

**Figure 1:**
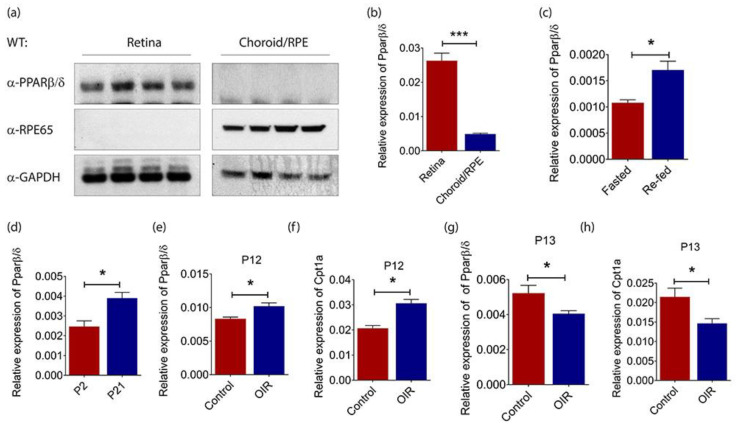
Peroxisome proliferator-activated receptor (PPAR)β/δ is highly expressed in mouse retina. (**a**) Representative Western blot of PPARβ/δ, retinal pigment epithelium-specific protein 65 kDa (RPE65) and glyceraldehyde-3-phosphate dehydrogenase (GADPH) in the retina (*n* = 4) and choroid/RPE (*n* = 4) compartments of adult C57BL/6 mice. Relative gene expression of *Pparβ/*δ in (**b**) the retina (*n* = 10) and choroid/RPE (*n =* 3) compartments of C57BL/6 mice; (**c**) the retina of fasted (*n =* 4) and re-fed (*n* = 4) adult C57BL/6 mice; (**d**) the retina of P2 (*n* = 4) and P21 (*n =* 4) C57BL/6 mice, as determined by quantitative real-time polymerase chain reaction (RT-qPCR) analysis. Relative expressions of (**e**) *Pparβ*/*δ* and (**f**) Carnitine palmitoyltransferase 1A (*Cpt1a*) in P12 hyperoxic retina of C57BL/6 mice subjected to oxygen-induced retinopathy (OIR) (*n =* 4) as compared to those in age-matched normoxic retina (*n =* 4), as determined by RT-qPCR analysis. Relative expressions of (**g**) *Pparβ*/*δ* and (**h**) *Cpt1a* in P13 hypoxic retina of C57BL/6 mice subjected to OIR (*n =* 4) as compared to those in age-matched normoxic retina (*n =* 4), as determined by RT-qPCR analysis. Gene expressions are quantified relative to the housekeeping gene, *Gadph*, as detailed in the Materials and Methods. Data are expressed as mean ± standard error of the mean (SEM). Unpaired, two-tailed t-test was used for statistical analysis; *** *p* < 0.001, * *p* < 0.05.

Genetic disruption of *Pparβ*/*δ* and specific PPARβ/δ antagonists have previously been shown to attenuate laser-induced CNV in mice [21]. Surprisingly, our study showed no detectable PPARβ/δ protein in the choroid/retinal pigment epithelium (RPE) fraction of C57BL/6 mouse eyes (Figure 1a). Retinal pigment epithelium-specific protein 65 kDa (RPE65) was only observed in the choroid/RPE fraction, verifying the isolation procedure. Consistently, retinal *Pparβ*/*δ* mRNA level was around 5-fold higher than that in choroid/RPE compartment (Figure 1b). Furthermore, the gene expression of *Pparβ*/*δ* remained unchanged in both retina (Figure S1a) and choroid/RPE (Figure S1b) compartments of C57BL/6 mice subjected to laser-induced CNV. Considering the high PPARβ/δ levels in normal mouse retina, an essential role of PPARβ/δ in fatty acid oxidation (FAO) [22,23], and the emerging role of FAO in EC metabolism and angiogenesis [13], we went on exploring the association between retinal *Pparβ*/*δ* expression and changes in the body’s metabolic status. C57BL/6 mice were subjected to overnight fasting followed by refeeding with normal chow diet. Our study showed that retinal *Pparβ*/*δ* level was significantly higher in refed mice as compared to that in mice undergoing fasting (Figure 1c), which confirmed what was previously seen in liver and kidney [24]. The mouse retina is avascular at birth and the retinal vasculature is fully established within the first few weeks of life [25]. As FAO is an aerobic process, we next analyzed the expression of *Pparβ*/*δ* in fully vascularized postnatal day (P)21 retina and compared it to that in avascular P2 retina. As expected, *Pparβ*/*δ* was expressed at a much lower level in avascular hypoxic retina at P2 (Figure 1d). 

The mouse model of oxygen-induced retinopathy (OIR) is able to reliably reproduce the defining characteristics of retinopathy of prematurity in human. Exposing P7 pups to 75% of oxygen for 5 continuous days not only causes the regression of immature retinal vessels but also prevents the formation of normal retinal vasculature [26]. The resulting central avascular retina becomes hypoxic once the mice are returned to room air at P12 [26]. *Pparβ*/*δ* expression was highly induced in the retina of mice subjected to 5 days of hyperoxia treatment as compared to that in age-matched C57BL/6 mice that had been kept under normoxic condition during this period (Figure 1e). Carnitine palmitoyltransferase 1A (*Cpt1a*) is a rate-limiting enzyme for FAO, and it has been reported to regulate angiogenesis [27]. *Cpt1a* is also a well-established target of PPARβ/δ [28]. Our data showed that *Cpt1a* was significantly induced in the retina of mice exposed to hyperoxia (Figure 1f). Similar to what we had observed in the developing retina, *Pparβ*/*δ* expression in hypoxic mouse retina at P13 was significantly lower than that in the retina of age-matched control mice that had been kept under normoxic condition (Figure 1g). As expected, *Cpt1a* expression was also suppressed in hypoxic mouse retina (Figure 1h). Together, these data showed a tight nutritional and environmental regulation of retinal *Pparβ*/*δ* levels.

### 2.2. Pparβ/δ Deficiency Does Not Affect Retinal Angiogenesis

**Figure 2:**
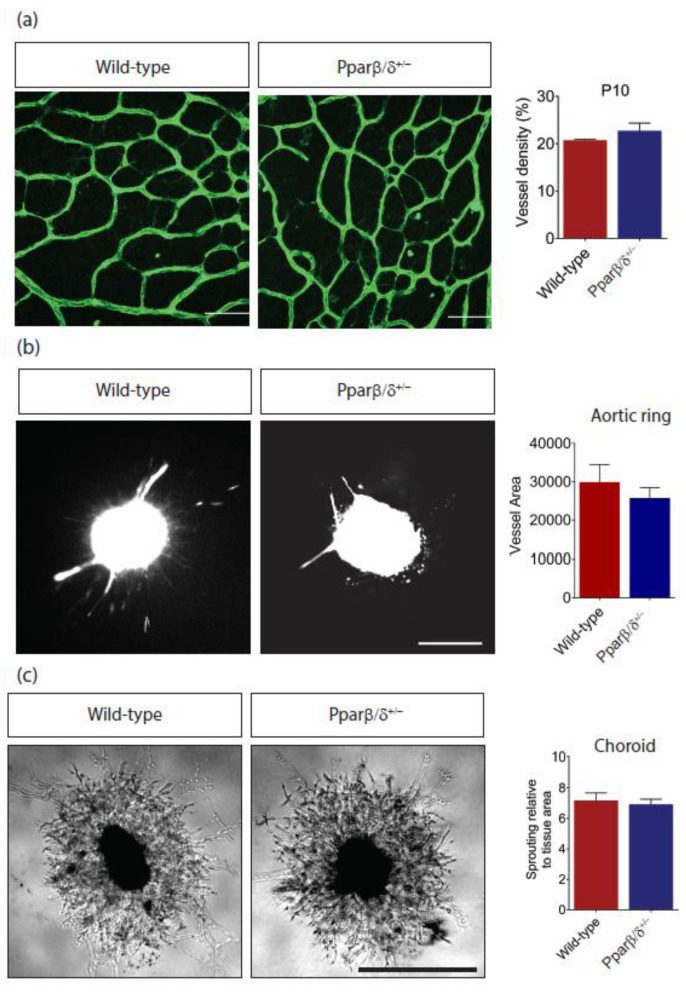
*Pparβ*/*δ* deletion does not affect normal blood vessel formation. (**a**) Representative images and quantification of retinal vessel density of P10 *Pparβ/δ^+/−^* (*n =* 4) and wild type (*n =* 3) retina. Scale bar: 50 µm. (**b**) Representative images and quantitative analysis of vessel outgrowth from P10 aortic rings isolated from *Pparβ/δ^+/−^* (*n* = 10 explants) and wild-type littermate controls (*n =* 5 explants). Scale bar: 200 µm. (**c**) Representative images and quantitative analysis of vessel outgrowth from P3 choroid explants isolated from *Pparβ/δ^+/−^* (*n =* 49 explants) and wild-type littermate controls (*n =* 34 explants). Scale bar: 200 µm. Data are expressed as mean ± SEM. Unpaired, two-tailed *t*-test was used for statistical analysis.

Placental defects were reported in *Pparβ*/*δ*^−/−^ mice, which leads to 90% midgestation lethality [29,30]. In our hand, around 18% of *Pparβ*/*δ*^−/−^ mice survived into adulthood. *Pparβ*/*δ*-haplodeficient mice were used in most cases to study the functional role of PPARβ/δ in retinal angiogenesis because their phenotype corresponded to some human retinal diseases (see below). Retinal vasculature was visualized in posterior eyecup whole mounts by immunofluorescence staining with a vascular endothelial cell marker, CD31. Quantitative analysis showed no difference in vascular density between *Pparβ/δ^+/−^* and wild-type littermate controls (Figure 2a). Next, to mimic new blood vessel formation in vivo, aorta rings were prepared from P10 *Pparβ/δ^+/−^* and wild-type littermate controls. There was no difference in vessel outgrowth from explanted aortic rings between *Pparβ/δ^+/−^* and wild-type counterparts (Figure 2b). This observation was further supported using choroidal angiogenesis assay in which vessel outgrowth from *Pparβ/δ^+/−^* and wild-type choroid explants were analyzed and showed no difference (Figure 2c). 

**Figure 3:**
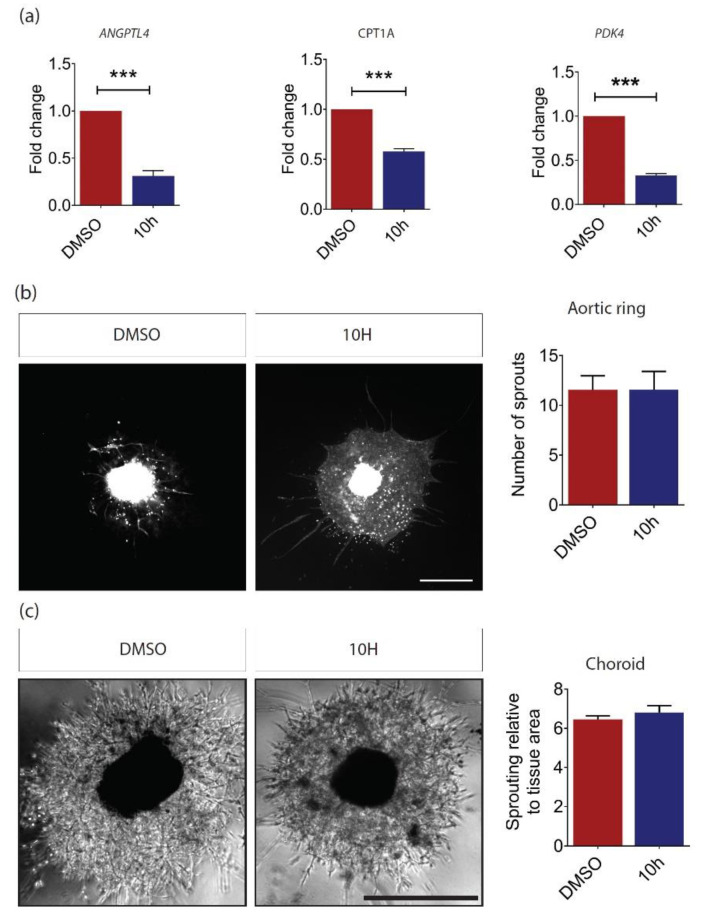
PPARβ/δ inhibition by 10h does not affect angiogenesis. (**a**) Relative gene expression of Angiopoietin-like 4 (*ANGPTL4*)*, CPT1A*, and Pyruvate Dehydrogenase Kinase 4 (*PDK4*) in human retinal microvascular endothelial cells (ECs) treated with 100 nM of 10h for 24 h compared to that in dimethyl sulfoxide (DMSO) vehicle control, as determined by RT-qPCR analysis (*n =* 3). (**b**) Representative images and quantitative analysis of vessel outgrowth from P3 C57BL/6 aortic ring explants treated with 100 nM of 10 h (*n =* 9 explants) and DMSO control (*n =* 11 explants). Scale bar: 200 µm. (**c**) Representative images and quantitative analysis of vessel outgrowth from P3 C57BL/6 choroidal explants treated with 100nM of 10h (*n =* 15 explants) and DMSO control (*n =* 34 explants). Scale bar: 200 µm. Data are expressed as mean ± SEM. Unpaired, two-tailed t-test was used for statistical analysis; *** *p* < 0.001.

To confirm these observations, wild-type aortic rings and choroid explants were treated with a novel PPARβ/δ antagonist, 10h, which specifically antagonizes the agonist-mediated transcriptional activity of PPARβ/δ and not the ligand activation of the two other PPAR isotypes [31]. The 10h-mediated PPARβ/δ inhibition was confirmed by evaluating the expression of PPARβ/δ target genes in human retinal microvascular ECs. Our results demonstrated a significant reduction in Angiopoietin-like 4 (*ANGPTL4*), *CPT1A*, and Pyruvate Dehydrogenase Kinase 4 (*PDK4*) gene expression levels (Figure 3a). However, no change in vessel outgrowth was observed in 10h-treated aortic rings (Figure 3b) and choroid explants (Figure 3c) as compared to those treated with dimethyl sulfoxide (DMSO) vehicle control. Together, our data showed that normal blood vessel formation was not affected by *Pparβ*/*δ*-haplodeficiency and in the presence of the specific PPARβ/δ antagonist 10h.

### 2.3. PPARβ/δ Mediates Blood Vessel Remodeling

**Figure 4:**
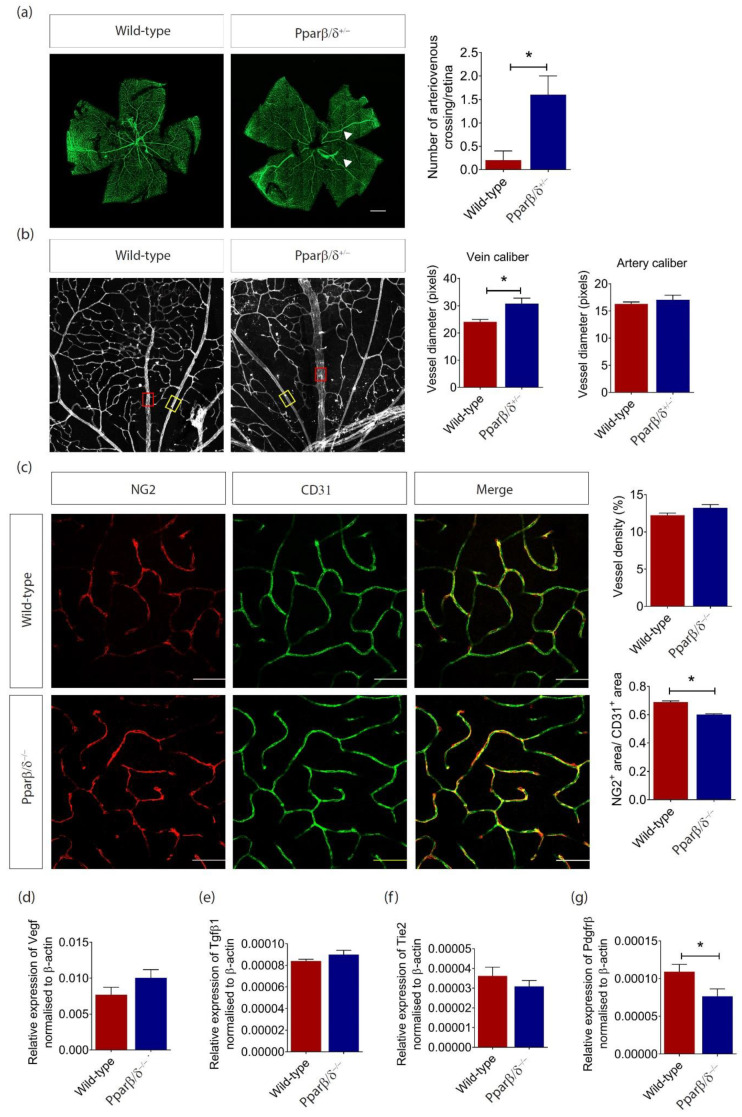
PPARβ/δ mediates retina blood vessel remodeling. (**a**) Representative retina images of flatmounted P10 *Pparβ/δ^+/−^* and wild type retinas, where arteriovenous crossings are highlighted by white arrowheads. Corresponding quantification of arteriovenous crossings in *Pparβ/δ^+/−^* (*n =* 4) and wild type (*n =* 5) retinas. Scale bar: 500 µm. (**b**) Representative images of the vein (red box) and artery (yellow box) in P10 *Pparβ/δ^+/−^* and wild type retinas. Corresponding quantification of the vessel diameter of the radial veins and arteries of *Pparβ/δ^+/−^* (*n =* 4) and wild type retinas (*n =* 5). Two points of at least 4 veins/arteries were measured per retina. (**c**) Representative confocal images for NG2 (red) and CD31 (green) staining of the retinal vasculature in adult *Pparβ/δ^−/−^* and wild type retinas. Scale bar: 50 µm. Corresponding quantification of the vessel density and pericyte coverage in wild type (*n =* 3) and *Pparβ/δ^−/−^* (*n =* 5) retinas. Relative gene expression of (**d**) *Vegf*, (**e**) *Tgfβ1*, (**f**) *Tie2*, and (**g**) *Pdgfrβ* mRNA of adult *Pparβ/δ^−/−^* and wild type retinas (*n* ≥ 3), as determined by RT-qPCR analysis. Data are expressed as mean ± SEM. Unpaired, two-tailed t-test was used for statistical analysis; * *p* < 0.05.

Although there was no difference in retinal vessel density between *Pparβ/δ^+/−^* and wild-type control mice at P10, a closer look at the retinal microvasculature revealed an increased incidence of crossover of the radial arteries and veins and of their side branches in the *Pparβ/δ^+/−^* mice (Figure 4a), a feature that is closely associated with branch RVO in human [3,32,33]. We further observed a wider caliber of radial veins but not radial arteries in *Pparβ/δ^+/−^* mice (Figure 4b), which is also observed in human patients with branch RVO [34]. Consistent with the observation in *Pparβ/δ^+/−^* mice, no change in retinal vessel density was observed in adult *Pparβ/δ^−/−^* mice; however, there was a significant reduction in pericyte coverage (Figure 4c). To support these observations, we investigated the expression of a panel of vascular markers that are involved in angiogenesis and blood vessel remodeling. As expected, there was no change in the expression of the genes for key angiogenic markers, including *Vegf* (Figure 4d), Transforming Growth Factor Beta 1 (*Tgfβ1*) (Figure 4e), and TEK Receptor Tyrosine Kinase (*Tie2*) (Figure 4f). However, the expression of Platelet Derived Growth Factor Receptor Beta Polypeptide (*Pdgfrβ*), a key molecule involved in pericyte recruitment and blood vessel remodeling, was significantly reduced in the retina of adult *Pparβ/δ^−/−^* mice as compared to that in wild-type controls (Figure 4g). Together, these data showed an important role of PPARβ/δ in the remodeling of retinal vasculature, and PPARβ/δ likely exerts its function through regulating PDGFRβ signaling.

### 2.4. PPARβ/δ Is Specifically Required for Pathological Retinal Angiogenesis

**Figure 5:**
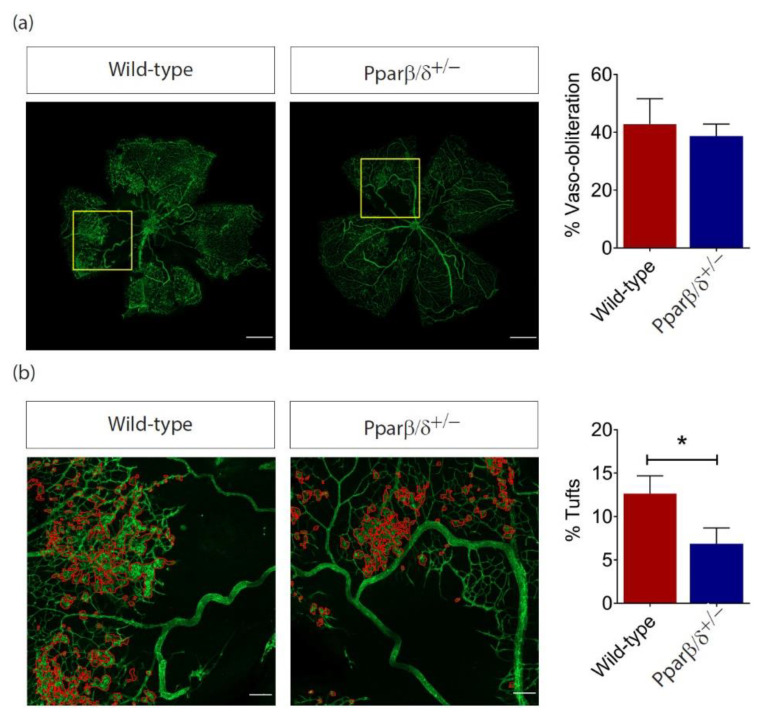
PPARβ/δ is specifically required for pathological retinal angiogenesis. (**a**) Representative immunofluorescence images of CD1 stained (green) retinal flatmount of P17 *Pparβ/δ^+/−^* and wild type retinas subjected to OIR. Scale bar: 500 μm. Corresponding quantification of retinal vaso-obliteration in P17 OIR retinas (*n =* 6). (**b**) Pathological neovascular tufts (delineated in higher magnification by red boundary line) and the corresponding quantification of tufts (normalized to retinal area) in *Pparβ/δ^+/−^* and wild type P17 OIR retinas (*n* ≥ 3). Scale bar: 100 μm. Data are expressed as mean ± SEM. Unpaired, two-tailed *t*-test was used for statistical analysis; * *p* < 0.05.

As our data thus far had demonstrated an altered *Pparβ*/*δ* expression in the retina under the hypoxic and hyperoxic conditions, we investigated whether intra-retinal/pre-retinal neovascularization, as observed in PDR, was affected in *Pparβ/δ^+/−^* mice subjected to OIR. Consistent with our observation in neonatal and adult mice, there was no significant difference in physiological neovascularization between *Pparβ/δ^+/−^* and wild-type animals as demonstrated by percentage of avascular area at P17 (Figure 5a). However, the area occupied by disordered neovascular growth (tufts), either as small isolated neovascular tissue or as large continuous areas lying on the surface of the retina, was significantly reduced in *Pparβ/δ^+/−^* mice (Figure 5b). This data suggests that PPARβ/δ was specifically required for disordered blood vessel formation in the mouse retina.

## 3. Discussion

Abnormal blood vessel formation is a defining feature of many blinding eye diseases, including PDR, nAMD, and RVO. Current targeted treatment options for these diseases focus on a single angiogenic pathway VEGF. However, a substantial number of patients are not responsive to the treatment or may develop resistance over time [35,36]. It is not surprising as angiogenesis is a highly complex and dynamic process involving extensive interactions between different types of cells, growth factors, and extracellular matrix components [37]. The inhibition of one angiogenic pathway may be compensated by the activation of alternative angiogenic pathways [5,6]. Furthermore, accelerated fibrovascular progression and potential neural toxicity associated with long-term anti-VEGF treatment pose a significant concern. There is an urgent need for alternative or complementary treatments to anti-VEGF therapeutics.

Although ECs are traditionally believed to rely on anaerobic glycolysis for adenosine triphosphate (ATP) production [38], FAO has been shown to modulate EC, especially stalk cell, proliferation, and angiogenesis [39]. Targeting EC metabolism may, therefore, offer an alternative strategy to control abnormal blood vessel formation. The PPAR transcription factors play key roles in cell metabolism [40], and all three PPAR isotypes are reported to regulate angiogenesis [17]. However, PPARβ/δ’s role in new blood vessel formation, especially those in the eye, is less well characterized.

A previous study reported a reduced lesion size in *Pparβ/δ^−/−^* mice subjected to laser-induced CNV [21]. Surprisingly, our study could not detect PPARβ/δ in the RPE/choroid fraction of C57BL/6 mouse eyes. Furthermore, the expression levels of PPARβ/δ was not affected in retina and choroid/RPE fractions of eyes subjected to laser-induced CNV. It is likely that PPARβ/δ activation instead of its expression is involved the development of CNV. Further experiments should be carried out to evaluate the expression of PPARβ/δ target genes, such as *Angptl4*, in laser-treated mouse eyes. As the focus of this study was PPARβ/δ, we have not explored the expression of other PPAR isotypes in the eyes following nutritional or hyperoxic challenges. However, hypoxia was reported to suppress the expression of PPARα in intestinal epithelial cells [41] and PPARγ in proximal renal tubular cells [42]. On the other hand, PPARα is induced in mouse heart and liver during fasting [43]. Based on these observations, PPARα and PPARγ are likely to be regulated by metabolic modifications in the eye.

Since PPARβ/δ was expressed in mouse retina at a relatively high level and its expression was affected by the body’s metabolic status, we went on to evaluate the role of PPARβ/δ in retinal angiogenesis. Our study suggests that *Pparβ*/*δ*-haplodeficiency had no impact on normal blood vessel formation as demonstrated in the retina of neonatal and adult mice, as well as in ex vivo angiogenesis assays. This observation was supported by treatment of wild-type aortic ring and choroid explants with the PPARβ/δ antagonist, 10h. In line with this observation, the expression of key angiogenic factors, including VEGF and TGFβ, were not affected in the retina of *Pparβ*/*δ*-null mice. Similar as in other vital organs, the arteries in the retina are responsible for taking oxygen and nutrient-rich blood to the retina, whereas veins carry the oxygen and nutrient depleted blood back to the lung and heart. Arteriovenous malformation disrupts this critical process. Although there was no change in retinal vascular density, *Pparβ/δ^+/−^* mice demonstrated an increased incidence of arteriovenous crossover in the retina, a feature that is commonly associated with hypertensive retinopathy and RVO in human [26,44,45]. Due to the low survival rate of *Pparβ/δ^−/−^* mice, *Pparβ*/*δ*-haplodeficient mice were used in most experiments for this study. To circumvent high lethality rate, mouse models with ocular cell-specific knockout of *Pparβ*/*δ* could be used to study the tissue-specific functions of PPARβ/δ.

We further observed an increase in venous caliber in *Pparβ/δ^+/−^* mice. Retina venous dilation is closely associated with a range of macro- and micro- vascular disorders, such as cerebrovascular diseases, including stroke [46,47,48,49], coronary heart disease [50,51], diabetic retinopathy, and diabetic nephropathy [46,52,53]. As changes in retinal vasculature could be visualized non-invasively and directly in vivo, and these changes are believed to serve as ‘markers’ of systemic disorders [54], it may be worth evaluating blood pressure, as well as the presence of other vascular complications, in PPAR-deficient mice in the future. Furthermore, external stimuli, such as hypoxia and oxidative stress, have been shown to contribute to the development of both macro-[55] and micro-[56] vascular complications. Considering the role of PPARβ/δ in ocular vessel remodeling, it is worth exploring its role in other vascular beds, including the heart and the brain. PPARβ/δ has previously been implicated in regulating vascular permeability [57]. In the retina, pericytes play a key role in maintaining blood vessel integrity and permeability. Our study demonstrated a reduced pericyte coverage in *Pparβ/δ^−/−^* retina. This observation was further supported by reduced gene expression of *Pdgfrβ*, which plays an important role in pericyte recruitment, in *Pparβ/δ^−/−^* retina. Nevertheless, future studies verifying the change of PDGFRβ at the protein level would be necessary to confirm this observation. The role of PPARβ/δ in pericyte signaling and function, pericyte-EC interaction, as well as the crosstalk between PPARβ/δ and PDGFRβ, also warrants further investigation. Finally, *Pparβ*/*δ* deletion in host tissue specifically inhibits the formation of pathological neovascularization tufts but has no impact on normal blood vessel regrowth. Previous studies indicate different metabolic profile in physiological and pathological vessels [58]. Considering an important role of PPARβ/δ in energy metabolism, it will be interesting to see whether it is involved in the metabolic switch associated with the progression of pathological neovascularization. Together, our data demonstrated a critical role of PPARβ/δ in retinal blood vessel remodeling and pathological angiogenesis.

## 4. Materials and Methods

### 4.1. Animals

*Pparβ*/*δ*-null mice (mixed genetic background of Sv129/C56BL/6) were kind gifts from Prof. Walter Wahli (Nanyang Technological University, Singapore; University of Lausanne, Switzerland). *Pparβ/δ^+/−^, Pparβ/δ^−/^*^−^, and wild-type controls (*Pparβ/δ^+/^*^+^) used in this study were offspring produced from haplodeficient heterozygous breeding pairs. *Pparβ/δ^+/^*^−^ maintained on a regular chow diet. For experiments with mutant mice, we used *Pparβ/δ^+/−^* mice, except for the pericyte study in which *Pparβ/δ^−/−^* were analyzed, because of availability due to difficult crossing. C57BL/6 mice were purchased from the InVivos Pte Ltd., Singapore. All animal procedures were reviewed and approved by the Nanyang Technological University Institutional Animal Care and Use Committee (IACUC, Project number: A18092, Approved: 15 Jan 2019), Singapore, and Institutional Animal Care and Use Committee of Agency for Science, Technology and Research (A*STAR) (IACUC, Protocol number: 181334). Genotyping was performed for the *Pparβ/δ^+/−^, Pparβ/δ^−/^*^−^, and wild-type controls (*Pparβ/δ^+/^*^+^) using polymerase chain reaction (PCR), which was performed using DreamTaq Hot Start Green PCR Master Mix (2×) (Thermo Fisher Scientific, Waltham, MA, USA), according to manufacturer’s instructions. Sequencing primers are listed in Table 1. Representative genotype result is shown in Figure S2.

### 4.2. Cell Culture

Human retinal microvascular ECs (HRMECs) (Angio-Proteomie, Boston, MA, USA) were cultured in endothelial growth medium (EGM) (Angio-Proteomie, USA) containing 10% fetal bovine serum (FBS), recombinant growth factors, and 1× penicillin and streptomycin. Cells were grown on Quick Coat Solution (Angio-Proteomie, USA) coated plates and maintained in humidified 5% CO_2_ /95% air at 37 °C.

### 4.3. Drug Treatment

PPARβ/δ antagonist, 10h (or methyl 3-(N-(4-(isopentylamino)-2-methoxyphenyl)sulfamoyl)-thiophene-2-carboxylate) [31,59], was a gift from Walter Wahli (Nanyang Technological University, Singapore; University of Lausanne, Switzerland) and Shunsuke Chiba (Nanyang Technological University, Singapore). DMSO (0.1%, *v*/*v*) (Thermos Fisher Scientific, USA) was used as a vehicle control. Recombinant Human VEGF_165_ was purchased from PeproTech (Rocky Hill, NJ, USA).

### 4.4. RNA Extraction and Quantitative Real-Time PCR

Mouse retinal and choroid/RPE tissues were immediately dissected after enucleation and were snap frozen in liquid nitrogen. Total RNA was extracted from homogenized mouse tissues using NucleoSpin RNA, Mini kit (Macherey-Nagel, Düren, Germany) following manufacturers’ instructions. cDNA was synthesized using qScript cDNA Supermix (Quanta BioSciences, Beverly, MA, USA) according to manufacturer’s instructions. Quantitative real-time PCR was performed in a total volume of 20 µL containing PrecisionFAST 2× qPCR Mastermix (with SYBR green and low ROX) (Primerdesign, Camberley, UK) using a QuantStudio™ 6 Flex Real-Time PCR System (Thermo Fisher Scientific, USA). Primers sequences used in this study are listed in (Table 2). Primers utilized for human *ANGPTL4, CPT1A*, and *PDK4* were PrimeTime qPCR primers from Integrated DNA Technologies, where their Primer Assay IDs were Hs.PT.58.25480012, Hs.PT.58.2799026, and Hs.PT.58.28212793, respectively. Except for where the expression of *Pparβ/δ* in the mouse retina and choroid/RPE was normalized to *β-actin*, all gene expressions were quantified relative to *Gapdh* in the quantitative real-time polymerase chain reaction (RT-qPCR) analysis.

### 4.5. Protein Extraction and Western Blot Analysis

Snap frozen mouse retinal and choroid/RPE tissues were homogenized in RIPA buffer containing 1mM dithiothreitol (DTT) (Sigma-Aldrich, St. Louis, MO, USA), 1× protease inhibitor (Roche, Switzerland), 1× phosphatase inhibitor (Sigma-Aldrich, USA), and 1mM phenylmethylsulfonyl fluoride (PMSF) (Sigma-Aldrich, USA) before being centrifuged at 13,000 rpm for 10 min at 4 °C. The supernatants were subjected to Bio-Rad Protein Assay (Bio-Rad, Hercules, CA, USA) for total protein analysis before being separated via sodium dodecyl sulfate-polyacrylamide gel electrophoresis (SDS-PAGE) and transferred onto an Immobilon-PSQ 0.2 µm Polyvinylidene Fluoride (PVDF) Membrane (Merck Millipore, Burlington, MA, USA). Blots were probed with PPAR delta antibody (rabbit polyclonal, ab8937, Abcam, Cambridge, UK, RRID:AB_306872), GAPDH antibody (rabbit polyclonal, sc-25778., Santa Cruz Biotechnology, Inc., Dallas, TX, USA, RRID:AB_10167668), or RPE65 antibody (mouse monoclonal, ab13826, Abcam, UK, RRID:AB_2181006), followed by horseradish peroxidase (HRP)-conjugated secondary antibodies (Bethyl Laboratories, Inc., Montgomery, TX, USA).

### 4.6. Mouse Model of Laser-Induced Choroidal Neovascularization

CNV was induced in wild type mice as described [60], and the eyes were harvested from the mice 21 days post laser. The retinae were immediately dissected and snap frozen for gene expression analysis.

### 4.7. Mouse Model of Oxygen-Induced Retinopathy (OIR)

OIR was induced as described [61]. In brief, P7 mice and their nursing mothers were placed in a 75% oxygen supply chamber for 5 days and exposed to a standard 12 h light-dark cycle. Mice were returned to room air at P12, and the retinae were collected at P17 for immunohistochemistry. The retinae for gene expression analysis were harvested at P12 (immediately after removal of mice from the hyperoxic chamber) and at P13 (24 h after return to room air).

### 4.8. Retina Flat Mount Immunohistochemistry

The eyes were rapidly enucleated and fixed in 4% paraformaldehyde (PFA) for 2 min before being dissected in flower shape in 2× phosphate-buffered saline (PBS). The retinae were fixed again in 100% methanol and stained with primary antibodies against CD31 (rat monoclonal, 553370, BD Biosciences, San Jose, CA, USA, RRID:AB_394816) and NG2 (rabbit polyclonal, AB5320, Merck Millipore, Germany, RRID:AB_11213678) overnight at 4 °C, followed by incubation with Alexa Fluor 488 or Alexa Fluor 594 secondary antibodies (A-11006 or A-11012, Thermo Fisher Scientific, USA) and 4′,6-diamidino-2-phenylindole (DAPI) (Thermo Fisher Scientific, USA). The retinae were flatmounted in ProLong Diamond Antifade Mountant (Thermo Fisher Scientific, USA) and examined by confocal microscopy (Zeiss LSM 800, Zeiss, Germany). The retinal vasculature was analyzed using AngioTool [62]. Pericyte coverage was determined by the ratio of NG2^+^ area/CD31^+^ area.

### 4.9. Aortic Ring Assay

The aortic ring assay was performed as described [60]. Aorta from P10 wild type and *Pparβ/δ^+/−^* mice were cut into 1mm rings before being embedded in a 96-well plate coated with rat tail collagen I gel (BD Biosciences, USA). Explants were treated with 50ng/mL VEGF and the media changed every other day. Explants from P3 wild type aorta were treated with 100nM of 10h or DMSO vehicle control. At day 10 of culture, the explants were fixed in 4% PFA and stained with Griffonia Simplicifolia isolectin B4 (IB4) (Vector Lab, Burlingame, CA, USA). Vessel outgrowth was imaged using Eclipse Ti-E Inverted Research Microscope (Nikon, Tokyo, Japan). The IB4^+^ areas were analyzed using ImageJ software (National Institutes of Health, Bethesda, MD, USA).

### 4.10. Choroid Sprouting Assay

The choroid sprouting assay was performed as described [63] using P3 mice. Choroid explants were treated with 100 nM 10 h or vehicle control. After three days, vessel outgrowth was imaged using Eclipse Ti-E Inverted Research Microscope (Nikon, Japan) and quantified using ImageJ software (National Institutes of Health, USA).

### 4.11. Statistical Analysis

All data are shown as mean ± SEM. Four independent repeats were carried out for each experiment unless stated otherwise. One-way ANOVA followed by Turkey’s post hoc analysis or unpaired two-tailed Student’s *t*-test was used to determine the statistical significance using GraphPad Prism 6 (GraphPad Software, San Diego, CA, USA). * *p* < 0.05; ** *p* < 0.01; *** *p* < 0.001; **** *p* < 0.0001.

## 5. Conclusions

While the deletion or inhibition of PPARβ/δ did not affect normal blood vessel formation, PPARβ/δ appeared to be specifically required for disordered blood vessel formation in the retina. *Pparβ*/*δ*-haplodeficiency was sufficient to cause an increased arteriovenous crossover and a wider venous caliber in neonatal mouse retina. Finally, complete removal of *Pparβ*/*δ* leads to reduced pericyte coverage in adult mice retina. Taken together, these findings highlight a critical role of PPARβ/δ in pathological angiogenesis and blood vessel remodeling in the retina.

## Figures and Tables

**Table 1:** Sequencing primers used for genotyping.

Primer	Sequence (5′–3′)
PBX9	AGACAATGATGGTGTGCTCA
PBX10	GCAGCTGCTGCTCAGCTGCCTGC
Rev1	CCTGAGACAGACTGCGCA
UMS	GCTCCTGAAGTCCACAATTCACAGTCC

**Table 2:** Sequences of the forward and reverse primers used in study for gene expression analysis.

**Target (Mouse)**	**Forward Sequence (5′–3′)**	**Reverse Sequence (5′–3′)**	**Amplicon Size (bp)**
*Pparβ/δ* (NM_011145.3)	CGGCAGCCTCAACATGG	AGATCCGATCGCACTTCTCATAC	143
*Cpt1a* (NM_013495.2)	AACACCATCCACGCCATACTG	TCCCAGAAGACGAATAGGTTTGAG	75
*Pdgfrb* (NM_001146268.1)	AGGAGTGATACCAGCTTTAGTCC	CCGAGCAGGTCAGAACAAAGG	152
*Vegfa* (NM_001025250.3)	TAGAGTACATCTTCAAGCCG	TCTTTCTTTGGTCTGCATTC	199
*Tek (Tie2)* (NM_013690.3)	GGCATTCCAGAACGTGAGAGAA	GATCCGGATTGTTTTTGGCCT	89
*Tgfb1* (NM_011577.2)	TTGCTTCAGCTCCACAGAGA	TGGTTGTAGAGGGCAAGGAC	183
*Actb (β-actin)* (NM_007393.5)	CCTTCTTGGGTATGGAATCCTGT	CACTGTGTTGGCATAGAGGTCTTTAC	101
*Gapdh* (NM_001289726.1)	ACTGAGGACCAGGTTGTCTCC	CTGTAGCCGTATTCATTGTCATAC	134
**Target (Human)**	**Forward Sequence (5′–3′)**	**Reverse Sequence (5′–3′)**	**Amplicon Size (bp)**
GAPDH (NM_002046.7)	GGTCTCCTCTGACTTCAACA	AGCCAAATTCGTTGTCATAC	116

## Supplementary Materials

Supplementary materials can be found at https://www.mdpi.com/1422-0067/21/12/4403/s1.

## Abbreviations

PPARβ/δ	Peroxisome proliferator-activated receptorβ/δ
CNV	Choroidal neovascularization
OIR	Oxygen-induced retinopathy
VEGF	Vascular endothelial growth factor
PDR	Proliferative diabetic retinopathy
DME	Diabetic macular edema
nAMD	Neovascular age-related macular degeneration
RVO	Retinal vein occlusion
PPARs	Peroxisome proliferator-activated receptors
ECs	Endothelial cells
RPE	Retinal pigment epithelium
kDa	kilodalton
RPE65	Retinal pigment epithelium-specific protein 65 kDa
FAO	Fatty acid oxidation
Angptl4	Angiopoietin-like 4
Cpt1a	Carnitine palmitoyltransferase 1A
GAPDH	Glyceraldehyde-3-phosphate dehydrogenase
RT-qPCR	Quantitative real-time polymerase chain reaction
Pdk4	Pyruvate dehydrogenase kinase 4
DMSO	Dimethyl sulfoxide
µm	micrometer
nM	nanomolar
Tgfβ1	Transforming Growth Factor Beta 1
Tie2	TEK Receptor Tyrosine Kinase
Pdgfrβ	Platelet Derived Growth Factor Receptor Beta Polypeptide
ATP	adenosine triphosphate
PCR	Polymerase chain reaction
DTT	dithiothreitol
PMSF	phenylmethylsulfonyl fluoride
SDS-PAGE	sodium dodecyl sulfate-polyacrylamide gel electrophoresis
PVDF	Polyvinylidene Fluoride
HRP	Horseradish peroxidase
PFA	paraformaldehyde
DAPI	4′,6-diamidino-2-phenylindole
PBS	phosphate-buffered saline
IB4	isolectin B4
