# Supplementary material for: Investigating the Role of PPARβ/δ in Retinal Vascular Remodeling Using Pparβ/δ-Deficient Mice
Source: Int J Mol Sci. 2020 Jun 20;21(12):4403. doi: 10.3390/ijms21124403 (PMC7353058; doi:10.3390/ijms21124403)
Supplement: Supplementary file 1 [file ijms-21-04403-s001.pdf]

Supplementary data

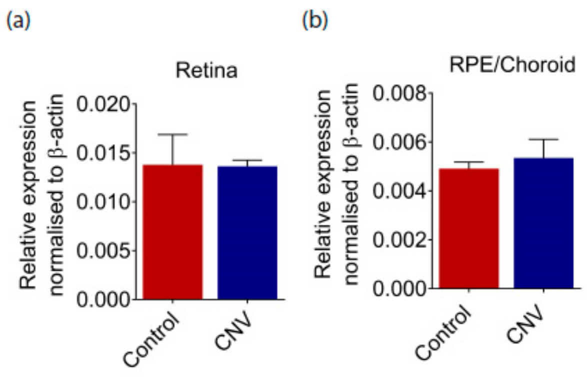

Figure S1. There is no change in *Pparβ/δ* expression in the retina and choroid/RPE fraction of the eye subjected to laser-induced choroidal neovascularization (CNV). (**a**) Relative gene expression of *Pparβ/δ* in wild type mouse retina subjected to laser-induced CNV (n = 3) and age-matched controls (n = 2), as determined by RT-qPCR analysis. (**b**) Relative gene expression of *Pparβ/δ* in wild type mouse choroid/RPE subjected to laser-induced CNV (n = 2) and age-matched controls (n = 3), as determined by RT-qPCR analysis. Data are expressed as mean ± SEM. Unpaired, two-tailed t-test was used for statistical analysis.

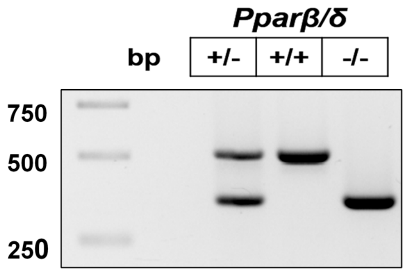

Figure S2. Genotyping of *Pparβ/δ*-deficient mice. Representative agarose gel showing genotyping data for *Pparβ/δ* heterozygous (+/−), wildtype (+/+), and null (−/−) mice. The *Pparβ/δ* wildtype (+) allele is represented by a band size of ~500bp, and the knockout (−) allele by a band size of ~350bp.
